# Impact of individual, household, and area characteristics on health and social care outcomes for people with multimorbidity: Protocol for a multilevel analysis

**DOI:** 10.1371/journal.pone.0282867

**Published:** 2023-10-05

**Authors:** Clare MacRae, Stewart W. Mercer, Andrew Lawson, Alan Marshall, Jamie Pearce, Eleojo Abubakar, Chunyu Zheng, Marjan van den Akker, Thomas Williams, Olivia Swann, Louisa Pollock, Anna Rawlings, Rich Fry, Ronan A. Lyons, Jane Lyons, Amy Mizen, Chris Dibben, Bruce Guthrie

**Affiliations:** 1 Advanced Care Research Centre, Bio Cube 1, Edinburgh BioQuarter, University of Edinburgh, Edinburgh, United Kingdom; 2 Usher Institute, College of Medicine and Veterinary Medicine, University of Edinburgh, Edinburgh, United Kingdom; 3 Department of Public Health Sciences, Medical University of South Carolina, Charleston, South Carolina, United States of America; 4 School of Geosciences, College of Science and Engineering, University of Edinburgh, Edinburgh, United Kingdom; 5 School of Social and Political Science, University of Edinburgh, Edinburgh, United Kingdom; 6 Institute of General Practice, Goethe University Frankfurt, Frankfurt am Main, Germany; 7 Department of Child Life and Health, University of Edinburgh, Edinburgh, United Kingdom; 8 Centre for Medical Informatics, Usher Institute, University of Edinburgh, Edinburgh, United Kingdom; 9 Child Health, School of Medicine, Dentistry & Nursing, University of Glasgow, Glasgow, United Kingdom; 10 Swansea University Medical School, Swansea, United Kingdom; UNITED STATES

## Abstract

**Background:**

Multimorbidity is one of the greatest challenges facing health and social care systems globally. It is associated with high rates of health service use, adverse healthcare events, and premature death. Despite its importance, little is known about the effects of contextual determinants such as household and area characteristics on health and care outcomes for people with multimorbidity. This study protocol presents a plan for the examination of associations between individual, household, and area characteristics with important health and social care outcomes.

**Methods:**

The study will use a cross-section of data from the SAIL Databank on 01 January 2019 and include all people alive and registered with a Welsh GP. The cohort will be stratified according to the presence or absence of multimorbidity, defined as two or more long-term conditions. Multilevel models will be used to examine covariates measured for individuals, households, and areas to account for social processes operating at different levels. The intra-class correlation coefficient will be calculated to determine the strength of association at each level of the hierarchy. Model outcomes will be any emergency department attendance, emergency hospital or care home admission, or mortality, within the study follow-up period.

**Discussion:**

Household and area characteristics might act as protective or risk factors for health and care outcomes for people with multimorbidity, in which case results of the analyses can be used to guide clinical and policy responses for effective targeting of limited resources.

## Introduction

Multimorbidity (often defined as two or more long-term conditions within an individual) is one of the greatest challenges facing health and social care systems globally [1]. This is because people with multimorbidity have higher rates of health service use [2], are more likely to experience adverse events associated with healthcare resulting from complex treatment regimens and polypharmacy [3], and are more likely to die prematurely [4] than the general population. Multimorbidity is common, affecting approximately a quarter of people in England [2] and Scotland [5]. It is strongly associated with age, for example in the UK people aged 80 years and older have a much higher likelihood of multimorbidity than those aged 20–29 years with an odds ratio of 59.35 [6]. Therefore, continuing projected future global population ageing [7] and the increasing number of younger people being affected [8] is likely to result in very large increases in the prevalence of multimorbidity, placing pressure on services.

Despite the importance of multimorbidity and prominence in the current literature [9], and understanding of the importance of individual characteristics such as age, sex, and lifestyle factors on health and care outcomes [10, 11], little is known about the wider determinants of health including household and area characteristics in the context of multimorbidity. Where a person lives, and who a person lives with, might act as protective or risk factors for health and care outcomes. These effects could be associated with environmental factors, both built or natural, that characterise the household or local area in which a person resides, and the “household composition” or characteristics of the other people who live in the same household or area [12]. Existing studies have investigated the impact of the household composition on healthcare outcomes [13], for example through examining associations between the health status of co-residents and emergency hospital admission rates in two-person households [14, 15]. Other household characteristics are known to affect the use of health care services including financial resources available to co-habitants [16], household food security [17], household tenure [18] and the availability of household co-residents to provide care [19] and to reduce social isolation [16]. Similarly, characteristics of the areas in which people live might impact healthcare outcomes through differences such as access to green space [20], distance to and accessibility of health care [21], and rates of crime and violence [22].

Understanding the importance of household and area characteristics in relation to health and social care outcomes for people with multimorbidity is important to help target limited resources more effectively. To do this, data about individuals nested in households and households nested in areas must be handled in a way that recognises that they exist at multiple levels, and that shared experiences of individuals will reduce their statistical independence. Use of conventional linear and logistic regression models cannot account for the social processes operating at these different levels, resulting in over-reporting of effect sizes. Therefore, this study will use frequentist multilevel models that account for hierarchies in data and allow appropriate examination of covariates at different levels.

## Methods

### Study design and population

The study population will be all adults alive and registered with a SAIL contributing GP in Wales on 01 January 2019, stratified by the presence of multimorbidity (defined as two or more long-term conditions [LTCs]). For each individual, the presence of multimorbidity will be described using 47 LTCs selected from a list recommended by a recent international Delphi consensus study [23] (S1 Table). LTCs will be extracted using code lists from the HDR-UK Phenotype Library applied to primary care and hospital inpatient datasets, and laboratory results [24]. Existing code-lists were adapted to extract prescribing data for LTCs where this information is required [25]. Phenotype definition and look-back duration for the codes defining each of the conditions followed rules defined by Barnett et al [5] where possible. For the remaining conditions, inclusion criteria were agreed through discussion between authors CM, SWM, and BG. In certain cases, look-back durations varied within conditions to reflect the impact that living with the condition was likely to have on an individual (see S2 Table for explanation of rules and code lists used to define each LTC).

Characteristics of individuals and the households and areas in which they live, to be included as covariates within the analysis, will be clustered hierarchically: individuals will be nested in households, and households will be nested in areas. Use of multilevel models will allow partial pooling of the coefficients to accommodate this data structure. Examination of the variation in individual-level outcomes at higher level clusters (at household and area levels) through partitioning of the variance will explain the strength of associations at different levels [26].

Covariates at the household level will be integral (for example the number of residents living in the household, and whether or not a household co-resident has multimorbidity) and at area levels will be derived (for example the area mean score for income) [27]. If covariate effects vary across clusters, the model can attempt to explain such differences by using cross-level interactions. For example, to examine whether differences in health outcomes are associated with the presence or absence of a household co-resident with multimorbidity. A logit multilevel model will be used to analyse the relationships between variables measured at the different levels of the data structure, where the outcome will be any health or care outcome during the study follow-up period.

### Outcomes

Study outcomes will be derived through follow-up of health and social care outcomes during the period 01 January 2019 to 31 December 2019 inclusive, thereby examining data for outcomes prior to the SARS CoV-2 pandemic.

The model will estimate the probability that a person has an adverse health or social care outcome (separate models will be used for each) given the covariates (Table 1), including the following events measured as a binary outcome:

- Attendance at the emergency department (ED), defined as any attendance at the ED in the Emergency Department Data Set (EDDS).
- Any emergency hospital admission, defined as any emergency admission to hospital referred by a GP, central referral service, consultant clinic or domiciliary visit, NHS direct, via the ED, or any other means of emergency admission, retrieved from the Patient Episode Database for Wales (PEDW).
- Admission to a care home, defined as any local authority or private care home providing either personal or nursing care for residents from the Care Home Dataset (CARE).
- All-cause mortality obtained from the Annual District Death Daily (ADDD).

**Table 1:** Health and care outcomes (measured at individual level).

Covariate	Data type and description
Emergency department (ED) attendance during study follow-up period	Categorical: binary (yes or no)
Emergency hospital admission during study follow-up period	Categorical: binary (yes or no)
Care home admission	Categorical: binary (yes or no)
Mortality	Categorical: binary (yes or no)

### Other variables

Associations with outcomes will be examined for a range of individual, household, and area characteristics.

Individual characteristics will include age, sex, ethnicity, smoking status, alcohol consumption, body mass index, previous health care use, and multimorbidity status (Table 2).

**Table 2:** Individual characteristics (covariates).

Covariate	Data type and description
Age in years	Continuous
Sex	Categorical: men, women
Ethnicity	Categorical: Asian, Black, mixed, other, White
Smoking status	Categorical: ex-smoker, current smoker, never smoked
Alcohol use	Categorical: drinker above recommended level, drinker at recommended level, drinker unknown quantity, non-drinker
Body mass index	Categorical: normal, underweight, obese, overweight
Total number of long-term conditions (LTCs)	Continuous
Total number of physical health LTCs	Continuous
Total number of mental health LTCs	Continuous
Emergency department attendance in previous 1 year	Continuous
Emergency hospital admission in previous 1 year	Continuous

Household characteristics will include household size and household co-resident multimorbidity status (Table 3). Analysis of the significance of the presence of co-residents with multimorbidity will be examined using an offset function of the household size variable thereby creating an in-model composite measure. Household size will be calculated by including all inhabitants of any age who live in the same household at the study cross-section date. Environmental characteristics will include household measures of ambient greenness and access to publicly available greenspace. Specifically, ambient greenness will be defined as average Normalised Difference Vegetation Index (NVDI) within 300m of the household location and access to publicly available greenspace as the distance to nearest greenspace in metres. NDVI is calculated from satellite images using the red and near infrared (NIR) wavelengths. Chlorophyll absorbs visible light more readily than NIR for use in photosynthesis and a greater percentage of NIR is reflected than visible light. Therefore, the NIR band in satellite images represents areas containing chlorophyll with high values and those without having lower values. NDVI is normalised and therefore the range of NDVI values is between -1 and 1. Measurement of covariates at household level is possible due to opportunities to examine address-linked electronic health records in the SAIL Databank facilitated through linkage of unique individual and household identifiers using the Unique Property Reference Number (UPRN) [28]. UPRN is the UK standard unique address identifier for every addressable location in Great Britain that allows accurate identification of property and street location [29].

**Table 3:** Household characteristics (covariates).

Covariate	Data type and description
Number of household residents	Continuous
Household resident multimorbidity count	Continuous
Household resident mental-physical multimorbidity count	Continuous
Ambient greenness (300m radius of household)	Continuous between 0 to 1 (scale of NVDI with 0 = least and 1 = most)
Distance to nearest park	Continuous (metres)

Area characteristics will include the individual domains of the Welsh Index of Multiple Deprivation (WIMD) including decile scores for each lower super output area (LSOA; a geographic small area in England and Wales designed to improve reporting of small area statistics, mean population of 1500) [30] reporting on material and social aspects of deprivation within areas: income, employment, health (collinearity with outcomes will be checked for this variable), education, access to services, community safety, physical environment, and housing [31]. A Consumer Data Research Centre metric, the Residential Mobility Index, will also be used to define the proportion of households in each LSOA where there has been change in resident(s) during the year 2018 (Table 4). Variables are available as deciles that define lack of access to opportunities and resources, including material deprivation defined as having insufficient physical resources needed to sustain a standard of living, and social deprivation categorises how the area characteristics relate to an individual’s ability to participate in normal social life of a community [31]. Social and environmental determinants of health are linked to health outcomes, and exposures to these risks are unequally distributed depending on inequalities of socioeconomic conditions and social determinants which impact on environmental conditions [32], making exploration of these domains of deprivation an important area of study.

**Table 4:** Area characteristics (covariates).

Covariate	Data type and description
Welsh Index of Multiple Deprivation (WIMD)	Categorical (deciles): 1 = most deprived to 10 = least deprived
WIMD domain—Access to services	Categorical (deciles): 1 = most deprived to 10 = least deprived
WIMD domain—Community safety	Categorical (deciles): 1 = most deprived to 10 = least deprived
WIMD domain—Education	Categorical (deciles): 1 = most deprived to 10 = least deprived
WIMD domain—Employment	Categorical (deciles): 1 = most deprived to 10 = least deprived
WIMD domain—Physical environment	Deciles: 1 (most deprived) to 10 (least deprived)
WIMD domain—Housing	Deciles: 1 (most deprived) to 10 (least deprived)
WIMD domain—Health	Deciles: 1 (most deprived) to 10 (least deprived)
WIMD domain—Income	Deciles: 1 (most deprived) to 10 (least deprived)
Consumer Data Research Centre (CDRC) Residential Mobility Index	Continuous: 0–1 (proportion of “churn” of residential population, households that have changed within 2019)

### Management of missing or incomplete data

Data in electronic health records can be missing for various reasons, such as incomplete recording or where a person does not interact with a service [33]. It is anticipated that there will be missingness of data in this analysis and covariates such as smoking status and alcohol use might be particularly affected. We hypothesise that younger people without multimorbidity are in general less likely to interact with services and therefore may have fewer measurements of blood pressure or recordings of smoking status or alcohol consumption.

Missing values could simply be ignored while running regression analyses, however this might result in limitation of the information available for the analysis [26]. Proportions of missingness for each covariate will be examined using univariate regression, and where missingness exceeds a predefined threshold the missing values will be imputed. Benefits of this approach are retention of the full sample size; however this can result in bias where a single imputation strategy is used (through assuming the true value with certainty) [26]. If the assumption of Missing at Random is true [34], and is adaptable to hierarchically structured data, Multiple imputation using Multiple Imputation by Chained Equations will be used to overcome this problem. This process commonly involves imputing a minimum of five values for each missing value, representing the sampling variability, to create a new complete dataset using a random draw of these imputed values [35].

### Data analysis

The analysis will be performed using R and will begin by fitting single level regression models using lm() and glm() functions to display and understand the fits of each. A multilevel model will then be fitted using lmer() functions, allowing intercepts and slopes to vary [36].

Step 1: Fit a single-level logistic regression unconditional model for each health and care outcome.

The model is:

$${\text{y}}_{ijk}{\text{|P}}_{ijk}∼Bern\left({\text{P}}_{ijk}\right)$$

$$Logit\left({\text{P}}_{ij\text{k}}\right)=\beta +r_{ij\text{k}}$$

Where y_*ijk*_ is the outcome (for the *i* ^th^ person, in the *j* ^th^ household, in the *k* ^th^ area), P represents the probability of the outcome, β the intercept or mean (the overall rate across all individuals and households), and γ is the random effect or the confounding from individuals that differentiates them from other study participants. The right-hand side of the equation describes the Bernoulli distribution of 0 or 1 for the outcome for each probability of each type of health or care outcome described in each model. A logit model is needed to map the outcome into minus to plus infinity. This model will not include any covariates.

Step 2: Fit a three-level random intercepts variance components model.

The model is:

$$\text{Logit}\left({\text{P}}_{ij\text{k}}\right)=\;\beta_{0}\;+\;{\text{v}}_{\text{k}}\;+\;u_{j}\;+\;{\text{r}}_{i}$$

Where β_0_ represents the overall intercept, v_k_ the random effects for level 3 (area), u_j_ the random effects for level 2 (household), and r_i_ the random effects for level 1 (individual).

V_k_ is the random term at the level of the area (effect of living in the area), u_jk_ (effect of living in the household), and r is random effect model for the individual.

The variance in the random effects are:

$$v_{k}∼N\left(0,\sigma_{v}^{2}\right)$$

$$u_{j}∼\mathrm{N mathvariant="normal"}\left(0,\sigma_{u}^{2}\right)$$

$$r_{ij}∼N\left(0,\sigma_{r}^{2}\right)$$

All variance, v_k_, u_j_, and r_ij_, are presumed to have a normal distribution with a mean of 0 and variance $\sigma_{v}^{2}$, $\sigma_{u}^{2}$ and $\sigma_{r}^{2}$ respectively.

Step 3: Calculate two estimates of the intraclass correlation coefficients (ICC).

Variance will be partitioned in an empty model to estimate the ICC. This will provide the degree of correlation with outcome (P(θ)) between two individuals in one household (level 2), and between two households in one area (level 3).

This variance partitioned by the *u* (household) component is:

$$\overset{⏜}{ICCu}=\frac{\sigma_{u}^{2}}{\sigma_{k}^{2}+\sigma_{u}^{2}+\sigma_{r}^{2}}$$

Step 4: Calculate the likelihood ratio test.

This allows comparison between the three-level model (Step 2) with the corresponding single-level version (Step 1) of the same model through testing the between-group variance, the reduction in deviance statistic. The null hypothesis is that variance is equal to 0, and the alternative is that variance is >0.

Step 5: Final covariate selection and final modelling strategy for three-level model.

The population will be stratified by multimorbidity status and therefore model outputs will be produced for each population presented as cohorts with and without multimorbidity. All variables will be fit on their own in a univariate analysis and the strength of this univariate association will guide the order of adding variables into the multivariate adjusted model. Selection of the final covariates to include in the adjusted model will be guided by minimisation of the Akaike Information Criterion where covariates with significant univariate ORs will be included.

### Outputs

Data will be represented in tables including univariate ORs and adjusted or multivariate (aORs) including strength of association for each covariate, graphical illustration of the strength of associations in a forest plot, and ICC (representing the expected correlation between units at each level) for individuals (level 1), households (level 2), and areas (level 3).

### Patient and public (PPI) involvement

Patient and public involvement has been central to the conceptualisation and design of this work and will continue to be throughout the duration of the project. At the planning stages, the proposal was discussed with patient and public involvement group at the Wellcome Trust Clinical Research Facility at the Western General Hospital, Edinburgh. Further input has been provided as part of the Information Governance Review Panel review process, a pre-requisite for accessing data within the SAIL Databank, and subsequently in a public and patient feedback group session organised by the Advanced Care Research Centre (ACRC) Knowledge Exchange Coordinators.

### Ethical agreement

This project will use anonymised health and administrative data about the population of Wales that is held in the SAIL Databank. The project was reviewed by the SAIL IGRP, a panel that includes the Chair of the Wales National Research Ethics Service, Caldicott Guardians, experts in data governance and protection, and representatives of the public. Approval for this project was granted on 20 December 2021.

### Dissemination of project findings

Dissemination of findings from the project to the research community will involve publication in peer-reviewed journals and presentation at national and international conferences. The ACRC Knowledge Exchange Coordinators will provide support by publishing lay summaries on the ACRC and Usher Institute websites, and dissemination through public engagement events.

## Discussion

This study will investigate associations between individuals, households, and areas with important health and social care outcomes for people with multimorbidity. Frequentist multilevel modelling of administrative data for a large population from the SAIL Databank in Wales will be used to understand the relative importance of covariates at each level. This approach will provide a comprehensive examination of the factors that contribute to important health and social care outcomes for people with multimorbidity.

Strengths of the methodological approach include a large population size providing sufficient power to estimate associations with outcomes more precisely, including outcomes that are rare, such as admission to a care home. The study will use administrative data with comprehensive coverage of the Welsh population collected systematically therefore representing the whole population [37]. Analyses will include many covariates across three levels of the data hierarchy accounting for the additional complexity of the hierarchical or nested structure of the data and the associated non-independence of within-level observations.

Limitations will include challenges faced when identifying which individuals have multimorbidity. Classification of morbidities across research studies often involve different definitions and criteria, data sources, and coding systems that might change over time [37]. Risk mitigation for this issue will involve use of publicly available published code lists [38] to be listed in supplementary material to enable other researchers to reproduce analyses in their own context. The study population will include people registered with a SAIL contributing practice which might introduce bias, for example those who are not registered with a GP, and are therefore excluded from the study, might be those living in areas with higher levels of deprivation and might be more at risk of adverse outcomes such as mortality. However, GP electronic records will be used to identify multimorbidity status and other individual level characteristics providing additional granularity that is otherwise unavailable within secondary care datasets. Finally, multilevel models can separately estimate associations for covariates at group-level (individual, household, and area in this study), they cannot be interpreted causally using these observational data. Despite this, developing understanding of associations of these covariates with health and care outcomes in this way is an important first step before progressing to examining causality.

This study will develop understanding of characteristics that put people with multimorbidity, a group with established associations with high use of healthcare resources and poor patient prognosis [39], at risk of important health and social care outcomes. Results can be used in both clinical and policy contexts to allow targeting of limited resources to the people most likely to benefit from them.

## Supporting information

S1 Table Choice of long-term conditions in relating to those included by “measuring multimorbidity in research: A Delphi consensus study” (Ho et al, 2022) [23]. (DOCX)

S2 Table List of included long-term conditions (LTCs) including explanation of rules and code-lists used to define them. LTCs were chosen according to the process described in S1 Table. Rules for implementation of each LTC were derived by referencing the rules used by Barnett et al [5] where present. Decisions were made through consensus among clinical authors of the study (CM, SWM, BG). Prescribing code lists (used to define anxiety, asthma, bipolar affective disorder, depression, and epilepsy) were transcribed into Read v2 codes from CPRD @ Cambridge—Code Lists (GOLD) [40].
(DOCX)
