# Supplementary material for: Impact of individual, household, and area characteristics on health and social care outcomes for people with multimorbidity: Protocol for a multilevel analysis
Source: PLoS One. 2023 Oct 5;18(10):e0282867. doi: 10.1371/journal.pone.0282867 (PMC10553261; doi:10.1371/journal.pone.0282867)
Supplement: S1 Table — (DOCX) [file pone.0282867.s001.docx]

# Supplementary Table 1. Choice of long-term conditions in relating to those included by “Measuring multimorbidity in research: a Delphi consensus study”

**(Ho et al, 2022)[21]**

| **Body system as defined by Delphi study** | **Delphi “always include” conditions** | **Delphi “usually include” conditions** | **Inclusion (including aggregation into higher-level condition) or exclusion (and rationale)** |
| --- | --- | --- | --- |
| Cardiovascular system | Stroke | - | Included (as stroke AND transient ischaemic attack combined) |
|  | Coronary artery disease | - | Included |
|  | Heart failure | - | Included |
|  | Peripheral arterial disease | - | Included |
|  | - | Heart valve disorders | Included |
|  | - | Arrythmia | Included |
|  | - | Venous thromboembolic disease | Included |
|  | - | Aneurysm | Included |
|  | - | Hypertension | Included |
| Metabolic and endocrine disease | Diabetes | - | Included |
|  | Addison's disease | - | Included |
|  | Cystic fibrosis | - | Included |
|  | - | Thyroid disorders | Included |
| Respiratory disease | Chronic obstructive pulmonary disease | - | Included |
|  | Asthma | - | Included |
|  | - | Bronchiectasis |  |
| Neurological disease | Parkinson's disease | - | Included |
|  | Epilepsy | - | Included |
|  | Multiple sclerosis | - | Included |
|  | Paralysis | - | Included |
|  | - | Transient ischaemic attack | Included (as stroke AND transient ischaemic attack combined) |
|  | - | Peripheral neuropathy | Included |
|  | - | Chronic primary pain | Excluded: it was decided to exclude based on CPRD @ Cambridge^34^ prescribing code list where listed analgesics could result in double counting of conditions. It was deemed difficult to avoid with use of a broad range of analgesic medications used for mild to moderate pain, for example osteo-arthritis for paracetamol, codeine-containing compounds, or non-steroidal anti-inflammatory drugs, and cancer for strong analgesics such as morphine |
| Cancer | Solid organ cancers | - | Included (as cancer) |
|  | Haematological cancers (included as cancer) | - | Included (as cancer) |
|  | Metastatic cancers (included as cancer) | - | Included (as cancer) |
|  | - | Melanoma (included as cancer) | Included (as cancer) |
|  | - | Cerebral tumours that can cause disability | Excluded: it was decided to exclude based on difficulty in defining this population using code lists applied to routinely collected data |
| Mental and behavioural disorder | Dementia | - | Included |
|  | Schizophrenia | - | Included |
|  |  | Depression | Included |
|  |  | Bipolar disorder | Included |
|  |  | Drug or alcohol misuse | Included |
|  |  | Eating disorder | Included |
|  |  | Autism | Included |
|  |  | Post-traumatic stress disorder | Included |
| Musculoskeletal disease | Connective tissue disease |  | Included |
|  |  | Osteoarthritis | Included |
|  | - | Long term musculoskeletal problems due to injury | Excluded: our group decided to exclude due to difficulty in accurately coding and identifying which conditions cause long-term impairment |
|  | - | Osteoporosis | Included |
|  | - | Gout | Included |
| Urogential disorder | Chronic kidney disease |  | Included |
|  | End stage kidney disease (included as chronic kidney disease) | - | Included |
|  | - | Endometriosis | Included |
|  | - | Chronic urinary tract infection | Excluded: it was decided to exclude due to difficulty in accurately coding and identifying which conditions cause long-term impairment. Using antimicrobial prescribing other than trimethoprim and nitrofurantoin also presents difficulties because these agents are not specific to infections of the urinary tract and broader spectrum antimicrobial agents could be used to treat acute soft tissue and respiratory infections |
| Haematological disorder | - | Anaemia | Included |
| Eye disease | - | Vision impairment that cannot be corrected | Included |
| Ear disease | - | Hearing impairment that cannot be corrected | Included |
|  | - | Meniere's disease | Included |
| Infectious disease | HIV | - | Excluded: not accessible in SAIL Databank |
|  |  | Chronic Lyme disease | Excluded: it was decided to exclude due to difficulty in accurately coding and identifying which conditions cause long-term impairment |
|  | - | Tuberculosis | Included |
|  | - | Post-acute covid-19 (study pre-2020) | Study period pre-2020 |
| Congenital disease | - | Congenital disease and chromosomal abnormalities | Included |
| Digestive disease | Chronic liver disease | - | Included |
|  | Inflammatory bowel disease | - | Included |
|  | - | Chronic pancreatitis | Included |
|  | - | Peptic ulcer | Included |
